# Normal saline versus heparin for patency of central venous catheters in adult patients - a systematic review and meta-analysis

**DOI:** 10.1186/s13054-016-1585-x

**Published:** 2017-01-08

**Authors:** Lei Zhong, Hai-Li Wang, Bo Xu, Yao Yuan, Xin Wang, Ying-ying Zhang, Li Ji, Zi-mu Pan, Zhan-Sheng Hu

**Affiliations:** 1 Department of Intensive Care Units, The First Affiliated Hospital of Jinzhou Medical University, No. 2, The Fifth Section of Renmin Street, Guta, Jinzhou, 121000 Liaoning China; 2 Department of Obstetrics and Gynaecology, The First Affiliated Hospital of Jinzhou Medical University, Jinzhou, 121000 Liaoning China; 3 Jinzhou Medical University, Jinzhou, 121000 Liaoning Province China; 4 Xinxiang Medical University, Xinxiang, 453000 Henan Province China

**Keywords:** Normal saline, Heparin, Central venous catheters, Occlusion

## Abstract

**Background:**

Heparin saline (HS) is theoretically superior to normal saline (NS) for maintaining the patency of central venous catheters (CVCs), but the comparative efficacy of them remains controversial. The aim of this systematic review and meta-analysis was to assess the efficacy of NS versus HS in the maintenance of the patency of CVCs in adult patients.

**Methods:**

We searched PubMed, Embase and the Cochrane library databases. Randomized controlled trials (RCTs) evaluating the use of NS vs. HS to maintain the permeability of CVCs among adult patients were included in our meta-analysis. References of relevant papers were reviewed manually. No language restriction was applied. Non-human studies were excluded. Pooled relative risk (RR) was calculated using a Mantel-Haenszel random-effects model. We also performed subgroup analysis examining the effect of the duration of catheter placement on the outcome. All statistical tests were two-sided using a significance level of 0.05.

**Results:**

Ten RCTs involving 7875 subjects (with analysis at patient, catheter, lumen and line access level) were included in this meta-analysis. Whether in terms of pooled or local analysis (RR with 95% confidence interval spans 1), NS can be equally, if not more effective, in keeping the CVCs open. Of studies reporting secondary outcomes (maneuver needed, heparin-induced thrombocytopenia, haemorrhage, central venous thrombosis and catheter-related bloodstream infection), heparinised saline was shown not to be superior to non-heparinised solution. Subgroup analysis in patients with short vs long term CVC placement was consistent with the main outcome partly and in particular for maintenance of catheter patency in patients with a long-term placement i.e. >30 days, the RR was 0.97 (*n* = 6589; 95% CI = 0.76 to 1.23; *P* = 0.796). However, for patients in whom the catheter was in place for <30 days, the RR was 1.52 (*n* = 1286; 95% CI = 1.02 to 2.27; *P* = 0.041).

**Conclusions:**

Based on the results of this meta-analysis, HS is not superior to NS in reducing CVCs occlusion. But in the short term, the use of HS is slightly superior to NS for flushing catheters from a statistical point of view.

**Electronic supplementary material:**

The online version of this article (doi:10.1186/s13054-016-1585-x) contains supplementary material, which is available to authorized users.

## Background

Central venous catheters (CVCs) are widely utilized in clinical practice, especially in intensive care units (ICUs) [1]. These devices are inserted so as to enable the administration of fluids, blood products, medications, parenteral nutrition, and for the performance of dialysis and central venous pressure monitoring [2, 3]. Currently, there are four types of CVCs: non-tunneled, tunneled, peripherally nserted central catheters (PICCs) and totally implantable venous access devices (TIVADs) [4].

These central lines will remain in place for days or even weeks each time [5]. Prolonged use may result in catheter occlusion, which may give rise to a requirement for the catheter to be treated, removed or replaced. Inserting a new central line creates latent threats, which could lead to disrupted treatment, increasing morbidity, and greater spending on health care [5]. Generally, catheter obstruction can be defined as partial occlusion (inability to aspirate blood but ability to flush freely) or complete occlusion (inability to flush freely and withdraw blood). It is estimated that the occlusion rate is between 0% and 33% when using heparin saline (HS) solution [6, 7]. Factors leading to catheter obstruction can be generally classified into three categories: mechanical causes, drug/mineral precipitates and clot formation, which is the most common reason overall [8]. To avoid the risk of catheter occlusion, thrombosis and catheter-related bloodstream infection (CRBSI), proper catheter flushing and locking are always considered to be the primary intervention because of the effect of reducing blood reflux into the lumen [8, 9].

Unfractionated heparin is well-known for its anticoagulant activity. Thus, heparin is widely used to maintain the patency of CVCs [10]. Nonetheless, the efficacy of this practice has not been definitively shown. Moreover, the use of heparinised saline is associated with potential risks such as coagulation disorders, hypersensitivity reactions and heparin-induced thrombocytopenia (HIT) [11, 12]. Researchers have been looking for a safe alternative to heparin, such as isotonic saline, vitamin C, lepirudin, sodium citrate or polygeline, to improve this situation [13–16]. Especially important, if there was a suitable replacement for HS, that would be beneficial, especially for patients with contraindications to using HS.

There have been numerous publications in this field over the last few years, including a guideline [17], several trials [2, 10] and several reviews [5, 9], including a Cochrane Review [18]. Most of these studies indicate that normal saline (NS) is safe and efficacious in preventing catheter occlusion in adult populations with CVCs. The recent guideline concluded that routine flushing with NS is recommended. However, the Cochrane review showed that there is no clear evidence to indicate whether NS flushing is superior to flushing with HS solution.

CVC occlusion is a fairly common problem, but differences in methods of prophylaxis, diagnosis and treatment practices related to catheter lumen obstruction vary, perhaps as a result of a lack of appropriate clinical guidelines [19]. An evidence-based, standardized flushing protocol is required for CVCs in adults. We conducted a systematic review and meta-analysis to evaluate the clinical efficacy (benefits and harms) of NS flushes and HS flushes for prevention of CVC lumen occlusion in adult patients.

## Methods

Our systematic review and meta-analysis was conducted according to preferred reporting items for systematic reviews and meta-analyses (PRISMA) guidelines (Additional file 1) [20]. The PICO framework was applied to define the clinical question clearly (Additional file 2). The primary outcome was catheter occlusion. Secondary outcomes included: maneuver needed (patients who required catheter manipulation to maintain the patency of the lumen), HIT, haemorrhage, central venous thrombosis and CRBSI.

### Search strategy and selection criteria

We systematically searched PubMed, Embase and the Cochrane library databases from the inception to 28 September 2016, using the following terms: “Sodium Chloride”, “Saline Solution, Hypertonic”, “NaCl”, “Heparin”, “Catheterization, Central Venous”, “Randomized Controlled Trial”, etc. (Additional file 3). There was no restriction on language. We also reviewed bibliographies in the retrieved articles to identify additional relevant studies. Only clinical randomized controlled trials (RCTs) of NS flushing vs flushing with HS solution in adults were included. Exclusion criteria were (1) age <18 years, and (2) case reports, letters, reviews, case-control studies and cohort studies, or non-human studies.

### Data extraction

Data were independently extracted by three reviewers (ZL, YY and WX). The following information was abstracted from the included studies: Study ID, mean age (years), country origin, number of subjects (NS/HS), female (%), centre, ICUs, disease types, follow up (days), heparin concentration (IU/ml) and heparin volume (ml). Disagreements were resolved by consensus.

### Assessment of study quality

The quality of the individual studies was assessed based on the Cochrane handbook for systemic reviews of interventions [21].

### Assessment of risk of bias

We performed sensitivity analysis to assess the influence of a single study on the pooled effects. Simultaneously, we used a funnel plot for assessment of publication bias [22].

### Statistical analysis

The pooled effects were analyzed by relative risk (RR) with 95% confidence interval (CI) for dichotomous outcomes. Statistical heterogeneity among trials was quantitatively assessed with the X^2^ test, *P* values and the *I* ^*2*^ statistics [23]. We pooled data using Mantel-Haenszel random-effects models, which are more conservative in their estimations [24].

During the search process, there were four different kinds of subjects for analysis: patients (six studies), catheters (two studies), lumens (one study; multilumen CVCs) and line access (one study; flushing central lines before and after each use). Given this, our meta-analysis was to be analysed separately on the basis of definite features of units.

In light of the Ge and Schallom study [2, 25], the duration of catheter placement is classified as short-term (less than 3–4 wks) and long-term (months to years). Simultaneously, subgroup analysis based on the length of indwelling time was carried out to characterize possible sources of heterogeneity(e.g. <30 and >30 days). All statistical tests were two-sided using an α level of 0.05. This meta-analysis was conducted using Stata 12.0 software (StataCorp, College Station, TX, USA) and Review Manager Version 5.3.5 (available from http://tech.cochrane.org/revman/download).

## Results

### Search results and study characteristics

**Fig. 1:**
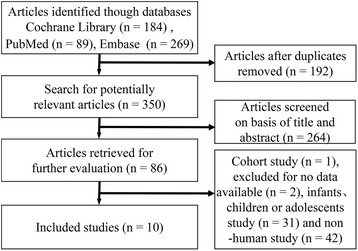
Flow chart of article selection procedure

The chart for the selection process and detailed information is given in Fig. 1. The literature search yielded 542 publications up to 28 September, 2016. We excluded 532 articles during the screening process. Thus, a total of 10 studies were eligible according to the inclusion criteria. The 10 studies were from Germany [13] (*n* = 1), Spain [6] (*n* = 1), Belgium [26] (*n* = 1), Italy [27] (*n* = 1), Japan [28] (*n* = 1), Iran [29, 30] (*n* = 2) and the USA [2, 31, 32] (*n* = 3). As a result, the 10 trials, including four ICU and six non-ICU studies, were reported in the years 2002–2015 and the average duration of follow up ranged from 1 to 400 days. The average age of the patients ranged from 51.6 to 68.43 years, and the proportion of female patients ranged from 31% to 65.34%. The different concentrations of heparin recorded in the existing publications ranged from 10 IU/mL to 5000 IU/ml.

In the Lyons trial [31], a comparison of 0.9% NaCl with two different concentrations of heparin (10 and 100U/ml) was presented. Consequently, we divided the data into two sets and performed statistical analysis. Ultimately, our study yielded 11 datasets from 10 RCTs. The baseline characteristics of the 10 eligible RCTs are shown in Table 1.

**Table 1:** Main characteristics of the included studies

Study ID	Mean age (years)	Country origin	Subjects, number (NS/HS)	Female (%)	Centre	ICUs	Disease types	Follow up (days)	Heparin concentration (IU/ml)	Heparin volume (ml)
Rabe 2002 [13]	59.25	Germany	33/33	63.64	S	Y	Multi-disease	20	5000	0.5
Kaneko 2004 [28]	68.43	Japan	26/22	50.00	S	N	Nephropathy	48	1000	2
Pumarola 2007 [6]	52.27	Spain	57/38	31.60	M	Y	Multi-disease	3	100	5
Bowers 2008 [32]	54.36	USA	50/52	50.00	S	N	Multi-disease	10.3	100	3
Schallom 2012 [2]	58.69	USA	150/145	48.81	S	Y	Multi-disease	14	10	3
Goossens 2013 [26]	55.81	Belgium	404/398	65.34	S	N	Cancer patients	180	100	3
Beigi 2014 [29]	63.1	Iran	49/47	45.83	S	N	Multi-disease	1	100	…
Lyons 2014 [31]	52	USA	28/30	40	H	N	Multi-disease	23	10	5
Lyons 2014 [31]	52	USA	28/32	40	H	N	Multi-disease	23	100	3
Molin 2015 [27]	62.69	Italy	203/212	53.49	M	N	Cancer patients	400	50	5
Ziyaeifard 2015 [30]	51.6	Iran	50/50	31.00	S	Y	Cardiac surgery	3	10	5

*S* single-centre study, *M* multi-centre study, *H* home care patient, *Y* yes, *N* no

### Assessment of study quality

**Fig. 2:**
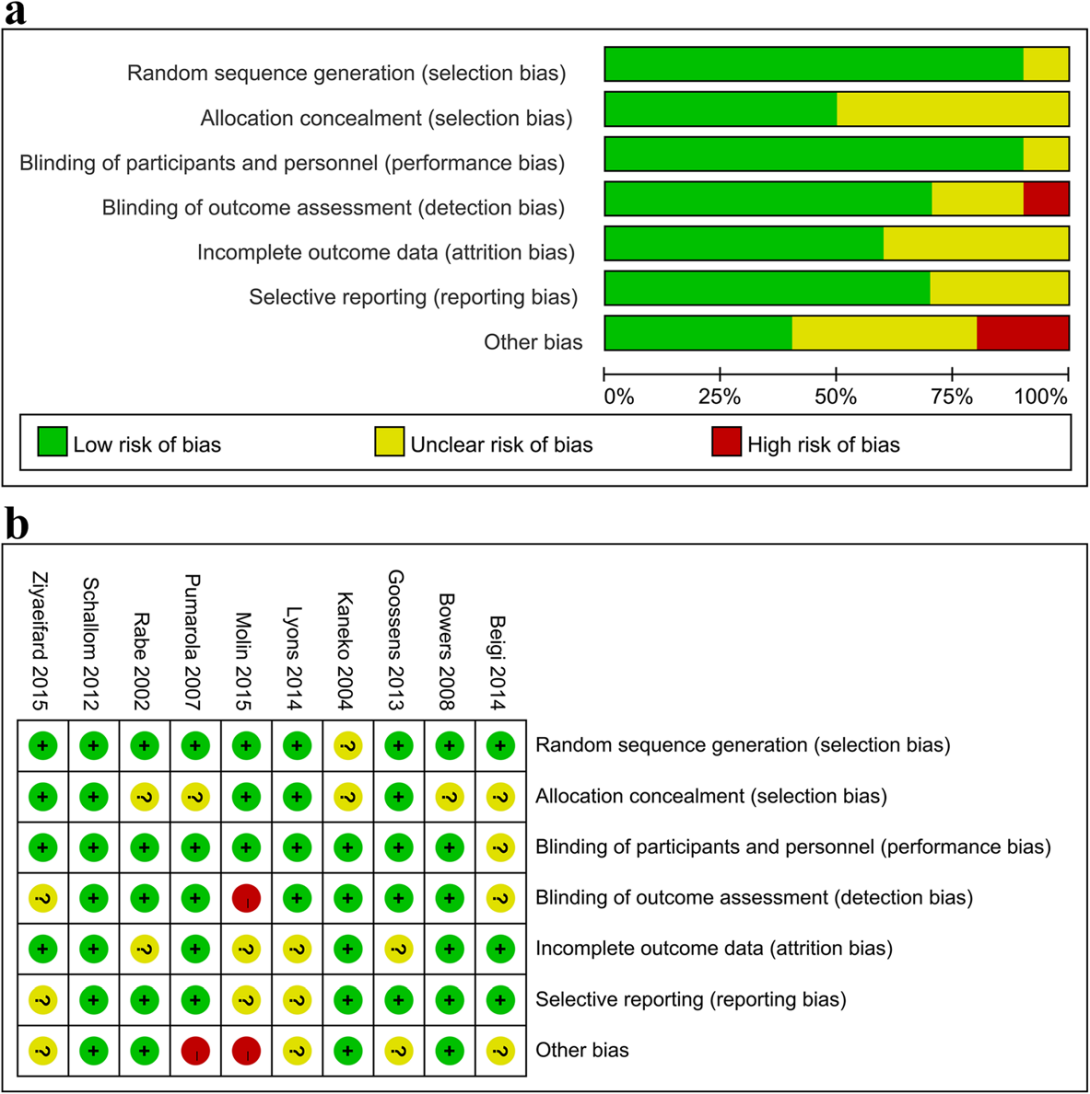
Risk of bias assessment. **a** Risks of bias graph. **b** Risks of bias summary

The risk of bias in the included studies is summarized in Fig. 2. It indicated that two papers had high risk of bias as they failed to obtain the expected sample size [6, 27], and one study was subject to detection bias, as the outcome measurement could have been influenced by lack of blinding [27].

### Assessment of reporting biases

**Fig. 3:**
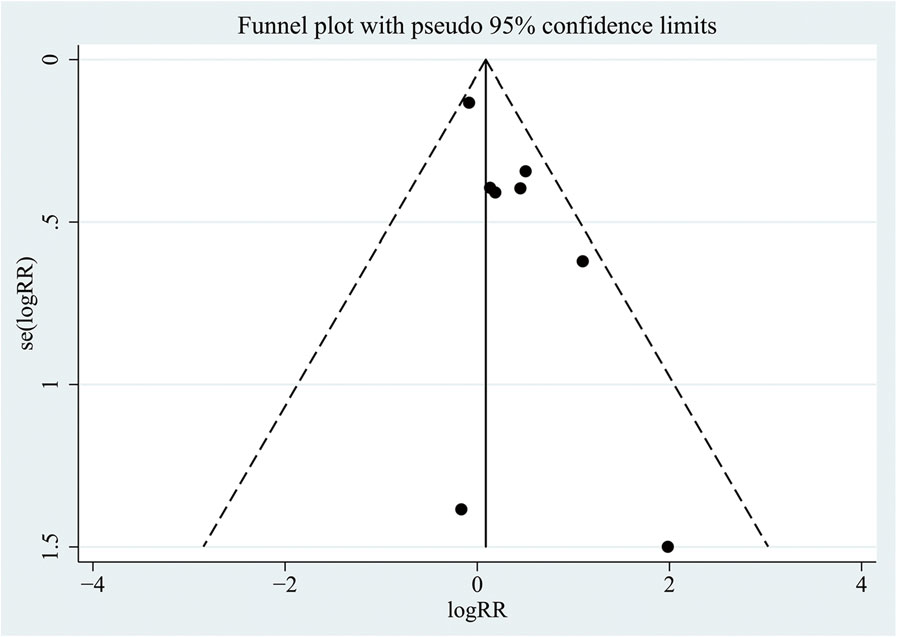
Funnel plot was generally asymmetrical. The *black dots* and *dotted line* indicate individual studies and 95% confidence intervals, respectively

We conducted sensitivity analysis, which suggested that the Goossens [26] study was the main source of statistical heterogeneity in our meta-analysis. However, we found it complied completely with the inclusion standards. Examination of the funnel plot suggests that there was also publication bias (Fig. 3).

The forest plot data prepared using Mantel-Haenszel random-effects models is summarized in Table 2.

**Table 2:** The summary results of all 10 studies

Result	Primary outcomes					Secondary outcomes					Subgroup analysis	
	Pooled effect	Catheter	Line access	Lumen	Patient	Maneuver needed	HIT	Haemorrhage	CVT	CRBSI	Short	Long
RR	1.21	3.00	0.92	1.66	1.33	1.24	1.33	0.75	0.81	0.84	1.52	0.97
95% CI lower-bound	0.91	0.89	0.71	0.85	0.86	0.71	0.09	0.32	0.50	0.11	1.02	0.76
95% CI upper-bound	1.61	10.10	1.19	3.24	2.07	2.16	18.54	1.74	1.31	6.71	2.27	1.23
*P* value	0.186	0.076	0.524	0.141	0.205	0.457	0.834	0.501	0.381	0.871	0.041	0.796

*HIT* heparin-induced thrombocytopenia, *CVT* central venous thrombosis, *CRBSI* catheter-related bloodstream infection, *RR* relative risk, *CI* confidence interval

### Synthesis of primary outcome

Pooled analysis was performed using a Mantel-Haenszel random-effects model and reported as RR with 95% CI (*n* = 7875; RR, 1.21; 95% CI = 0.91 to 1.61; *P* = 0.186), with a low heterogeneity among these studies (*X* ^2^ = 8.39, *P* = 0.299; *I* ^*2*^ = 16.6%).

**Fig. 4:**
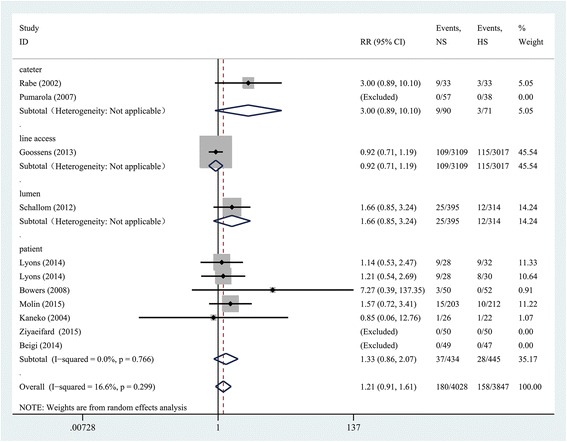
Forest plot of association between use of normal saline (NS) vs heparin saline (HS) and the incidence of catheter occlusion. *RR* relative risk, *CI* confidence interval, *NS* normal saline, *HS* heparin saline

From the catheter (*n* = 161; RR, 3.00; 95% CI = 0.89 to 10.10; *P* = 0.076), line access (*n* = 6126; RR, 0.92; 95% CI = 0.71 to 1.19; *P* = 0.524), lumen (*n* = 709; RR, 1.66; 95% CI = 0.85 to 3.24; *P* = 0.141) and patient (n = 879; RR, 1.33; 95% CI = 0.86 to 2.07; *P* = 0.205), HS is not superior to NS in preventing catheter occlusion (Fig. 4).

### Secondary outcomes

**Fig. 5:**
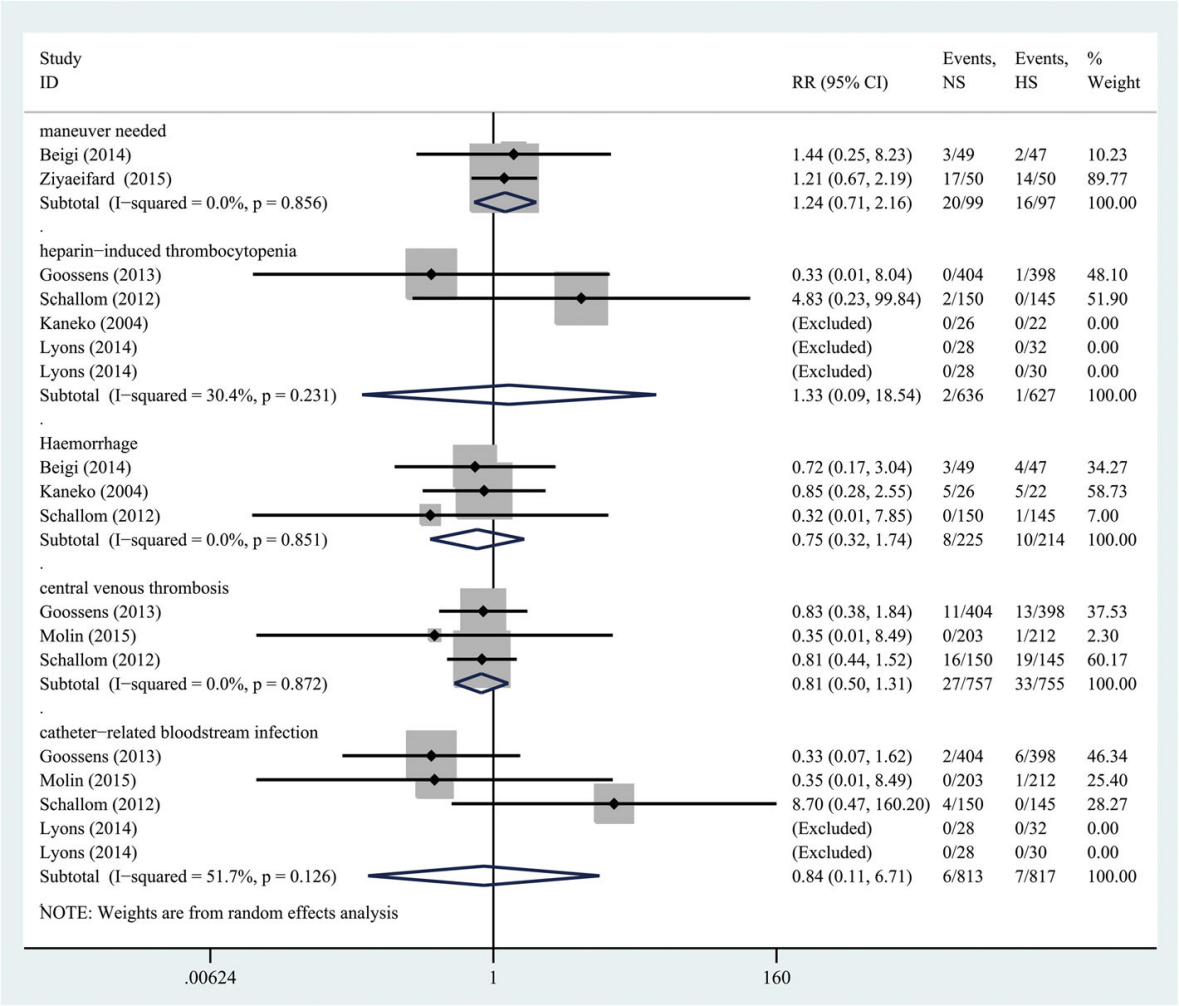
Forest plot of association between use of NS vs HS and the incidence of complications. *RR* relative risk, *CI* confidence interval, *NS* normal saline, *HS* heparin saline

For maneuver needed (*n* = 196; RR, 1.24; 95% CI = 0.71 to 2.16; *P* = 0.457), heparin-induced thrombocytopenia (*n* = 1263; RR, 1.33; 95% CI = 0.09 to 18.54; *P* = 0.834), haemorrhage (*n* = 439; RR, 0.75; 95% CI = 0.32 to 1.74; *P* = 0.501), central venous thrombosis (*n* = 1512; RR, 0.81; 95% CI = 0.50 to 1.31; *P* = 0.381) and catheter-related bloodstream infection (*n* = 1630; RR, 0.84; 95% CI = 0.11 to 6.71; *P* = 0.871), flushing CVCs with NS was as effective as HS (Fig. 5).

### The results of subgroup analysis

**Fig. 6:**
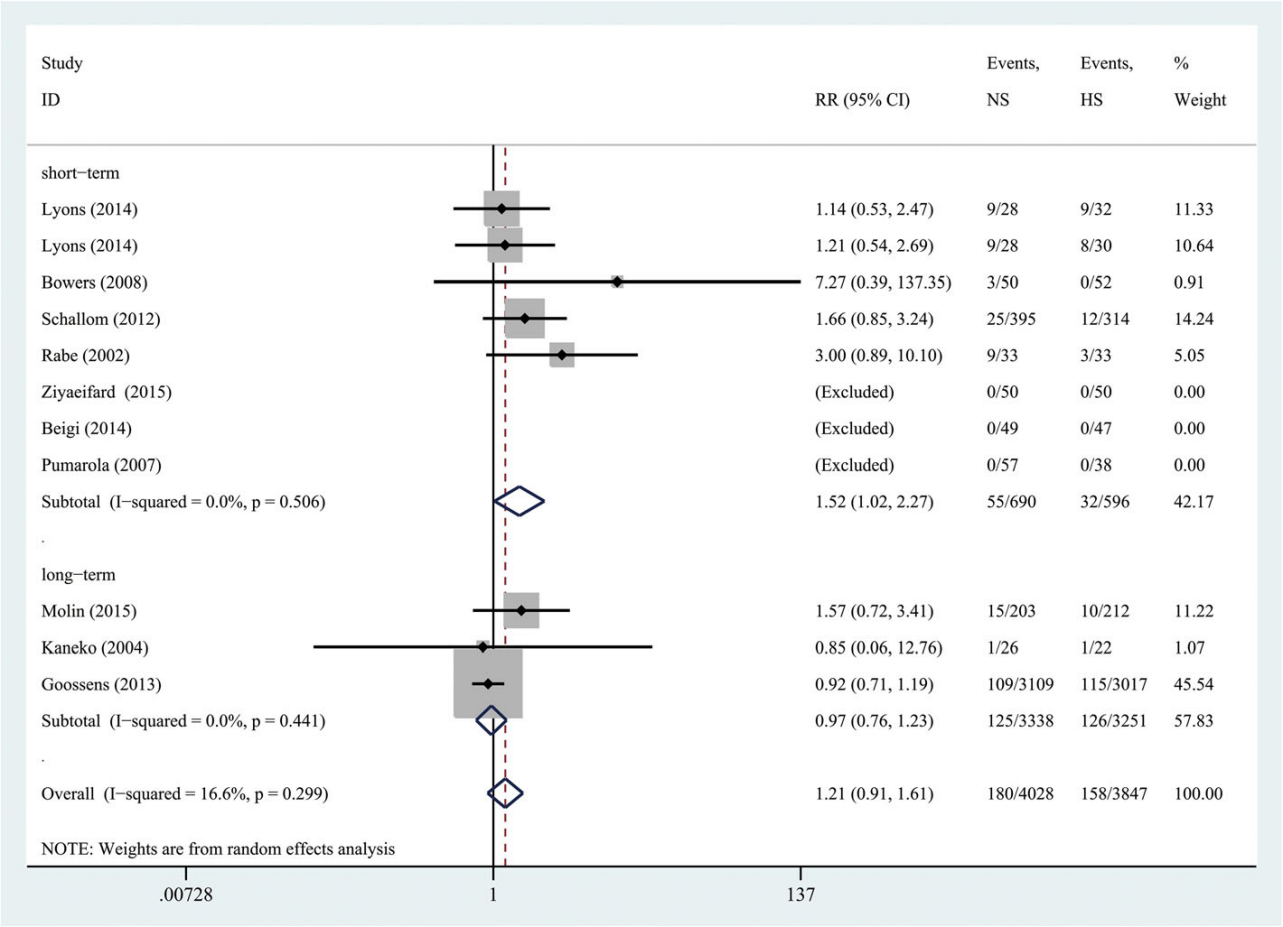
Subgroup analysis based on the duration of catheter placement. *RR* relative risk, *CI* confidence interval, *NS* normal saline, *HS* heparin saline

There were seven studies examining the duration of catheter placement at 30 days or less (*n* = 1286 subjects) and there were three studies examining duration of catheter placement of more than 30 days (*n* = 6589 subjects). Similar to the aforementioned, NS was equal in efficacy to using HS solution in long-term CVCs (*n* = 6589; RR, 0.97; 95% CI = 0.76 to 1.23; *P* = 0.796). By comparison, for short-term study, this analysis demonstrated that NS is less effective than HS (*n* = 1286; RR, 1.52; 95% CI = 1.02 to 2.27; *P* = 0.041) (Fig. 6).

## Discussion

Our systematic review and meta-analysis did not demonstrate a general difference between use of NS or HS in adult populations. In subgroup analysis, stratified by the length of indwelling time (e.g. <30 and >30 days), there appear to be two conflicting conclusions (Fig. 6.) In the short run (<30 days), HS was slightly better than NS. A plausible reason may be that NS has no anticoagulation activity. However, a marginally significant association was observed between using NS vs HS and the incidence of catheter occlusion. Owing to the limited numbers of included studies and the effect sizes, we should treat such a result with caution. By comparison, NS could be equal, if not more effective, in the long run (>30 days). This has implications for patients in whom long-term catheter use may be necessary, for example, in patients undergoing cancer treatment or those requiring dialysis. From a long-term perspective, the use of NS in these patients has several advantages over HS solutions. To begin with, NS is an isotonic solution, which is in accordance with basic physiological needs. In addition, the use of NS will result in fewer side effects from heparin-related complications. Finally, as HS is several times more expensive than NS [32], eliminating its use in flushing solutions has economic benefits.

To date, there have only been three relevant meta-analyses in this area (Additional file 4). The result of the first study (network meta-analysis) was no marked difference, when comparing adult patients with NS vs HS or other solutions in the flushing of CVCs [33]. The second study, consistent with results of previous research, found that HS was not more effective than NS in reducing catheter occlusion when analysed in three different areas (participant, catheter and line access) [18]. These findings challenged the continued use of HS in CVC flushing, as it is more expensive than saline solution. However, a recent study supported NS as a substitution for HS as a locking solution in CVCs in adult patients from the point of view of four different types of CVC [34]. Data from these studies suggests that HS may not be required to maintain the patency of CVCs. In the absence of sufficient evidence to support the use of NS, the debate will be moot. For this reason, further study is needed in this field.

To our knowledge, our study might be the first meta-analysis from the viewpoint of four different CVC-related areas (patient, catheter, lumen and line access) and indwelling time (i.e. <30 and >30 days). Our results, in accordance with current studies, meta-analyses and reviews [18, 27, 33], suggest that there is very little evidence to conclude that flushing with HS has more effect than NS flushing solution for CVC maintenance.

Only a few randomized controlled studies have compared NS with HS for maintenance of CVC lumen patency in adults. The Rabe study was the first RCT to compare the effects of NS versus HS and they determined that the use of a flush containing 5000 U/ml was more effective than NS [13]. In particular, catheter survival rate was higher in the HS group than in NS group. In contrast to the results of the Rabe study, a large number of other studies suggest that the catheter lumen occlusion rate is not different between those with vs those without heparin [10, 27–29]. NS flushing for CVCs has been applied in some American ICUs without supporting evidence [35]. Parallel with the mainstream view, Schiffer et al. [17] suggest that routine flushing of CVCs with NS to prevent occlusion is reasonable in this guideline. Morover, a report of a recent multicentre randomized trial [27] argued that HS was not more effective than NS in reducing withdrawal or total occlusion. No statistical difference in catheter patency was observed when comparing NS to HS solution.

Due to the body’s physiologic response to the catheters, nearly 100% of CVCs will develop a “fibrin sheath”, which may increase the risk of catheter occlusion from 1 to 14 days after insertion of the indwelling catheter [36, 37]. As the “fibrin sleeve” usually envelops the tip of the CVC, some argue that a heparin lock could not prevent thrombotic occlusion because of the difficulty in achieving an effective concentration on the outside of the catheter tip [28]. Our findings support these suggestions.

Certainly, the occurrence of CVC occlusion is related to the catheter type, puncture site, heparin concentration, heparin volume, flush frequency, retaining time and the patient’s physical condition [37, 38]. As there are indeterminate factors in this field, further studies, including well-designed trials, are warranted to assess these effects on clinical outcomes.

Various potential limitations should be taken into consideration. First, although the statistical heterogeneity was low, the clinical and methodological heterogeneity cannot be ignored. The latter two types of heterogeneity might be attributed to various types of participants, interventions, outcomes studied (partial or complete occlusion), study designs and study qualities. Second, the potential hazards might occur after long-term follow up, thus, some of these complications could be discarded due to the short duration of some included studies. Third, this meta-analysis was limited to studies conducted in Asia, Europe and North America, and thus, might not be generalizable to other parts of the world. Finally, there was a publication bias in our study as small studies with null results tend not to be published. Hence, uniform study design and multi-centre studies should be launched in different countries and regions to establish the best approach to long-term maintenance of CVCs.

## Conclusions

So far, there are still no criteria for flushing and locking techniques, volumes or regimens for safe CVC maintenance. In conclusion, this meta-analysis did not demonstrate any superiority of heparin locked saline solutions over NS for the maintenance of CVC lumen patency in adult patients. Thus, additional large prospective RCTs might be needed in this field due to the inconclusive evidence available.

## Key messages

- Few RCTs have compared NS to HS for prevention of catheter occlusion in adult patients.
- Pooling the results showed that flushing CVCs with NS is as effective as HS in adult patients, but the 95% confidence interval is wide and spans 1.
- There is a lack of evidence of the effectiveness of NS flushing compared to HS in keeping CVCs open.

## Additional files

Additional file 1: The PRISMA checklist. (DOC 64 kb)
Additional file 2: PICO framework. (DOCX 15 kb)
Additional file 3: The search strategy and search results. (DOCX 25 kb)
Additional file 4: Characteristics of the three relevant meta-analyses. (DOCX 37 kb)

## Abbreviations

CI: confidence interval
CRBSI: catheter-related bloodstream infection
CVCs: central venous catheters
HIT: heparin-induced thrombocytopenia
HS: heparin saline
ICUs: intensive care units
NS: normal saline
PICCs: peripherally-inserted central catheters
RCTs: randomized controlled trials
RR: relative risk
TIVADs: totally implantable venous access devices
