# Supplementary material for: Normal saline versus heparin for patency of central venous catheters in adult patients - a systematic review and meta-analysis
Source: Crit Care. 2017 Jan 8;21:5. doi: 10.1186/s13054-016-1585-x (PMC5219914; doi:10.1186/s13054-016-1585-x)
Supplement: Additional file 2: — PICO framework. (DOCX 15 kb) [file 13054_2016_1585_MOESM2_ESM.docx]

**Additional file 2: PICO framework.**

| **PICO element Study inclusion criterion** |
| --- |
| **Patients** Adult patients using central venous catheters  **Intervention** Normal saline flushing  **Comparison** Heparin saline flushing  **Outcomes** Catheter occlusion, maneuver needed, heparin-induced  thrombocytopenia, haemorrhage, central venous thrombosis,  catheter-related bloodstream infection |
