# Supplementary material for: Normal saline versus heparin for patency of central venous catheters in adult patients - a systematic review and meta-analysis
Source: Crit Care. 2017 Jan 8;21:5. doi: 10.1186/s13054-016-1585-x (PMC5219914; doi:10.1186/s13054-016-1585-x)
Supplement: Additional file 3: — The search strategy and search results. (DOCX 25 kb) [file 13054_2016_1585_MOESM3_ESM.docx]

**Additional file 3：The search strategy and search results**

PubMed (n=89), Embase (n=269) and the Cochrane library databases(n=184)

**PubMed:**

#1 (((((((((((NS[Title/Abstract]) OR NaCl[Title/Abstract]) OR sodium[Title/Abstract]) OR saline*[Title/Abstract]) OR Sodium Chloride, (24)NaCl[Title/Abstract]) OR Sodium Chloride, (22)Na[Title/Abstract]) OR Saline Solution[Title/Abstract]) OR "Sodium Chloride"[Mesh]) OR Sodium Chloride[Title/Abstract])) OR (((((((((((((((Solution, Hypertonic Saline[Title/Abstract]) OR Hypertonic Saline Solution[Title/Abstract]) OR Solutions, Hypertonic Saline[Title/Abstract]) OR Hypertonic Saline Solutions[Title/Abstract]) OR Sodium Chloride Solutions, Hypertonic[Title/Abstract]) OR Solution, Saline Hypertonic[Title/Abstract]) OR Saline Hypertonic Solution[Title/Abstract]) OR Hypertonic Solution, Saline[Title/Abstract]) OR Sodium Chloride Solution, Hypertonic[Title/Abstract]) OR Saline Solutions, Hypertonic[Title/Abstract]) OR Solutions, Saline Hypertonic[Title/Abstract]) OR Saline Hypertonic Solutions[Title/Abstract]) OR Hypertonic Solutions, Saline[Title/Abstract]) OR Saline Solution, Hypertonic[Title/Abstract]) OR "Saline Solution, Hypertonic"[Mesh])) **84483**

#2 ((((((((((((((((HS[Title/Abstract]) OR LMWH[Title/Abstract]) OR UFH[Title/Abstract]) OR UH[Title/Abstract]) OR hepar*[Title/Abstract]) OR alpha Heparin[Title/Abstract]) OR alpha-Heparin[Title/Abstract]) OR Heparin Sodium[Title/Abstract]) OR Heparin, Sodium[Title/Abstract]) OR Sodium Heparin[Title/Abstract]) OR Liquaemin[Title/Abstract]) OR Heparinic Acid[Title/Abstract]) OR Heparin, Unfractionated[Title/Abstract]) OR Unfractionated Heparin[Title/Abstract]) OR Heparin[Title/Abstract]) OR "Heparin"[Mesh]) **126933**

#3 ((((((((((Peripherally Inserted Central Catheter Line Insertion[Title/Abstract]) OR PICC Placement[Title/Abstract]) OR PICC Placements[Title/Abstract]) OR Placement, PICC[Title/Abstract]) OR PICC Line Placement[Title/Abstract]) OR PICC Line Placements[Title/Abstract]) OR peripherally inserted central catheters[Title/Abstract]) OR peripherally inserted central catheter[Title/Abstract]) OR PICC*[Title/Abstract])) OR ((((((((((((((((("Catheterization, Central Venous"[Mesh]) OR Catheterization, Central Venous[Title/Abstract]) OR Venous Catheterization, Central[Title/Abstract]) OR Central Catheterization[Title/Abstract]) OR Catheterization, Central[Title/Abstract]) OR Catheterizations, Central[Title/Abstract]) OR Central Catheterizations[Title/Abstract]) OR Catheterizations, Central Venous[Title/Abstract]) OR Central Venous Catheterizations[Title/Abstract]) OR Venous Catheterizations, Central[Title/Abstract]) OR Central Venous Catheterization[Title/Abstract]))) OR (((((Cannulations[Title/Abstract]) OR Cannulation[Title/Abstract]) OR Catheterizations[Title/Abstract]) OR Catheterization[Title/Abstract]) OR "Catheterization"[Mesh])) OR (((((((((((("Catheters, Indwelling"[Mesh]) OR Catheters, Indwelling[Title/Abstract]) OR Catheter, Indwelling[Title/Abstract]) OR Indwelling Catheter[Title/Abstract]) OR Indwelling Catheters[Title/Abstract]) OR In-Dwelling Catheters[Title/Abstract]) OR Catheter, In-Dwelling[Title/Abstract]) OR Catheters, In-Dwelling[Title/Abstract]) OR In Dwelling Catheters[Title/Abstract]) OR In-Dwelling Catheter[Title/Abstract]) OR Implantable Catheters[Title/Abstract]))) OR (((venous access device[Title/Abstract]) OR (((TIVAD*[Title/Abstract]) OR totally implanted venous access devices[Title/Abstract]) OR totally implanted venous access device[Title/Abstract])))) OR (((CVC*[Title/Abstract]) OR cannula*[Title/Abstract]) OR catheter*[Title/Abstract])) **320214**

#4 (random*[Title/Abstract]) OR ("Randomized Controlled Trial" [Publication Type] OR "Randomized Controlled Trials as Topic"[Mesh]) **1020239**

#5 #1 and #2 and #3 and #4 **89 (search results)**

**Search Name: NS vs. HS 2016. 2016.9.28 Cochrane library**

Last Saved: 28/09/2016 02:44:08.182

Description:

ID Search

#1 "Saline Solution, Hypertonic":ti,ab,kw or "Hypertonic Solutions, Saline":ti,ab,kw or "Saline Hypertonic Solutions":ti,ab,kw or "Solutions, Saline Hypertonic":ti,ab,kw or "Saline Solutions, Hypertonic":ti,ab,kw (Word variations have been searched) 468

#2 "Sodium Chloride Solution, Hypertonic":ti,ab,kw or "Hypertonic Solution, Saline":ti,ab,kw or "Saline Hypertonic Solution":ti,ab,kw or "Solution, Saline Hypertonic":ti,ab,kw or "Sodium Chloride Solutions, Hypertonic":ti,ab,kw (Word variations have been searched) 5

#3 "Hypertonic Saline Solutions":ti,ab,kw or "Solutions, Hypertonic Saline":ti,ab,kw or "Hypertonic Saline Solution":ti,ab,kw or "Solution, Hypertonic Saline":ti,ab,kw or "Sodium Chloride":ti,ab,kw (Word variations have been searched) 7075

#4 "Saline Solution":ti,ab,kw or "Sodium Chloride, (22)Na":ti,ab,kw or "Sodium Chloride, (24)NaCl":ti,ab,kw or "saline*":ti,ab,kw or "sodium":ti,ab,kw (Word variations have been searched) 40532

#5 "NaCl ":ti,ab,kw or "NS":ti,ab,kw (Word variations have been searched) 10049

#6 MeSH descriptor: [Saline Solution, Hypertonic] explode all trees 453

#7 MeSH descriptor: [Sodium Chloride] explode all trees 2310

#8 #1 or #2 or #3 or #4 or #5 or #6 or #7 **48994**

#9 "Heparin":ti,ab,kw or "Unfractionated Heparin":ti,ab,kw or "Heparin, Unfractionated":ti,ab,kw or "Heparinic Acid":ti,ab,kw or "Liquaemin":ti,ab,kw (Word variations have been searched) 8921

#10 "Sodium Heparin":ti,ab,kw or "Heparin, Sodium":ti,ab,kw or "Heparin Sodium":ti,ab,kw or "alpha-Heparin":ti,ab,kw or "alpha Heparin":ti,ab,kw (Word variations have been searched) 154

#11 "hepar* ":ti,ab,kw or " UH ":ti,ab,kw or "UFH":ti,ab,kw or " LMWH":ti,ab,kw or "HS":ti,ab,kw (Word variations have been searched) 11574

#12 MeSH descriptor: [Heparin] explode all trees 4361

#13 #9 or #10 or #11 or #12 **11850**

#14 "Catheterization, Central Venous":ti,ab,kw or "Venous Catheterization, Central":ti,ab,kw or "Central Catheterization":ti,ab,kw or "Catheterization, Central":ti,ab,kw or "Catheterizations, Central":ti,ab,kw (Word variations have been searched) 877

#15 "Central Catheterizations":ti,ab,kw or "Central Venous Catheterization":ti,ab,kw or "Catheterizations, Central Venous":ti,ab,kw or "Central Venous Catheterizations":ti,ab,kw or "Venous Catheterizations, Central":ti,ab,kw (Word variations have been searched) 958

#16 "Catheterization":ti,ab,kw or "Catheterizations":ti,ab,kw or "Cannulation":ti,ab,kw or "Cannulations":ti,ab,kw or "Catheters, Indwelling":ti,ab,kw (Word variations have been searched) 8686

#17 "Catheter, Indwelling":ti,ab,kw or "Indwelling Catheter":ti,ab,kw or "Indwelling Catheters":ti,ab,kw or "In-Dwelling Catheters":ti,ab,kw or "Catheter, In-Dwelling":ti,ab,kw (Word variations have been searched) 1351

#18 "Catheters, In-Dwelling":ti,ab,kw or "In Dwelling Catheters":ti,ab,kw or "In-Dwelling Catheter":ti,ab,kw or "Implantable Catheters":ti,ab,kw or "catheter*":ti,ab,kw (Word variations have been searched) 17260

#19 "cannula*":ti,ab,kw or "CVC*":ti,ab,kw or "totally implanted venous access devices":ti,ab,kw or "totally implanted venous access device":ti,ab,kw or "TIVAD*":ti,ab,kw (Word variations have been searched) 2633

#20 "venous access device":ti,ab,kw or "Peripherally Inserted Central Catheter Line Insertion":ti,ab,kw or "PICC Placement":ti,ab,kw or "PICC Placements":ti,ab,kw or "Placement, PICC":ti,ab,kw (Word variations have been searched) 92

#21 "Placements, PICC":ti,ab,kw or "PICC Line Placement":ti,ab,kw or "PICC Line Placements":ti,ab,kw or "Placement, PICC Line":ti,ab,kw or "Placements, PICC Line":ti,ab,kw (Word variations have been searched) 2

#22 "PICC Line Catheterization":ti,ab,kw or "Catheterization, PICC Line":ti,ab,kw or "Catheterizations, PICC Line":ti,ab,kw or "PICC Line Catheterizations":ti,ab,kw or "peripherally inserted central catheters":ti,ab,kw (Word variations have been searched) 103

#23 "peripherally inserted central catheter":ti,ab,kw or "PICC*":ti,ab,kw (Word variations have been searched) 232

#24 MeSH descriptor: [Catheterization, Central Venous] explode all trees 844

#25 MeSH descriptor: [Catheterization] explode all trees 9475

#26 MeSH descriptor: [Catheters, Indwelling] explode all trees 1027

#27 #14 or #15 or #16 or #17 or #18 or #19 or #20 or #21 or #22 or #23 or #24 or #25 or #26 **23055**

#28 "random*":ti,ab,kw or "trial":ti,ab,kw or "randomized controlled trial":ti,ab,kw (Word variations have been searched) 642188

#29 MeSH descriptor: [Randomized Controlled Trial] explode all trees 157

#30 #28 or #29 **642188**

#31 #8 and #13 and #27 and #30 **184 (search results)**

**Embase （269）**

Session Results

.......................................................

No. Query Results Results Date

#98. 'saline solution, hypertonic':ab,ti AND **269** 28 Sep 2016

([embase]/lim OR [medline]/lim) OR ('hypertonic

solutions, saline':ab,ti AND ([embase]/lim OR

[medline]/lim)) OR ('saline hypertonic

solutions':ab,ti AND ([embase]/lim OR

[medline]/lim)) OR ('solutions, saline

hypertonic':ab,ti AND ([embase]/lim OR

[medline]/lim)) OR ('saline solutions,

hypertonic':ab,ti AND ([embase]/lim OR

[medline]/lim)) OR ('sodium chloride solution,

hypertonic':ab,ti AND ([embase]/lim OR

[medline]/lim)) OR ('hypertonic solution,

saline':ab,ti AND ([embase]/lim OR

[medline]/lim)) OR ('saline hypertonic

solution':ab,ti AND ([embase]/lim OR

[medline]/lim)) OR ('solution, saline

hypertonic':ab,ti AND ([embase]/lim OR

[medline]/lim)) OR ('sodium chloride solutions,

hypertonic':ab,ti AND ([embase]/lim OR

[medline]/lim)) OR ('hypertonic saline

solutions':ab,ti AND ([embase]/lim OR

[medline]/lim)) OR ('solutions, hypertonic

saline':ab,ti AND ([embase]/lim OR

[medline]/lim)) OR ('hypertonic saline

solution':ab,ti AND ([embase]/lim OR

[medline]/lim)) OR ('solution, hypertonic

saline':ab,ti AND ([embase]/lim OR

[medline]/lim)) OR ('sodium chloride':ab,ti AND

([embase]/lim OR [medline]/lim)) OR ('saline

solution':ab,ti AND ([embase]/lim OR

[medline]/lim)) OR ('sodium chloride,

(22)na':ab,ti AND ([embase]/lim OR

[medline]/lim)) OR ('sodium chloride,

(24)nacl':ab,ti AND ([embase]/lim OR

[medline]/lim)) OR ('saline*':ab,ti AND

([embase]/lim OR [medline]/lim)) OR

('sodium':ab,ti AND ([embase]/lim OR

[medline]/lim)) OR ('nacl':ab,ti AND

([embase]/lim OR [medline]/lim)) OR ('ns':ab,ti

AND ([embase]/lim OR [medline]/lim)) OR 'sodium

chloride'/exp AND ('heparin':ab,ti AND

([embase]/lim OR [medline]/lim) OR

('unfractionated heparin':ab,ti AND ([embase]/lim

OR [medline]/lim)) OR ('heparin,

unfractionated':ab,ti AND ([embase]/lim OR

[medline]/lim)) OR ('heparinic acid':ab,ti AND

([embase]/lim OR [medline]/lim)) OR

('liquaemin':ab,ti AND ([embase]/lim OR

[medline]/lim)) OR ('sodium heparin':ab,ti AND

([embase]/lim OR [medline]/lim)) OR ('heparin,

sodium':ab,ti AND ([embase]/lim OR

[medline]/lim)) OR ('heparin sodium':ab,ti AND

([embase]/lim OR [medline]/lim)) OR

('alpha-heparin':ab,ti AND ([embase]/lim OR

[medline]/lim)) OR ('alpha heparin':ab,ti AND

([embase]/lim OR [medline]/lim)) OR

('hepar*':ab,ti AND ([embase]/lim OR

[medline]/lim)) OR ('uh':ab,ti AND ([embase]/lim

OR [medline]/lim)) OR ('ufh':ab,ti AND

([embase]/lim OR [medline]/lim)) OR ('lmwh':ab,ti

AND ([embase]/lim OR [medline]/lim)) OR

('hs':ab,ti AND ([embase]/lim OR [medline]/lim))

OR 'heparin'/exp) AND ('catheterization, central

venous':ab,ti AND ([embase]/lim OR [medline]/lim)

OR ('venous catheterization, central':ab,ti AND

([embase]/lim OR [medline]/lim)) OR ('central

catheterization':ab,ti AND ([embase]/lim OR

[medline]/lim)) OR ('catheterization,

central':ab,ti AND ([embase]/lim OR

[medline]/lim)) OR ('catheterizations,

central':ab,ti AND ([embase]/lim OR

[medline]/lim)) OR ('central

catheterizations':ab,ti AND ([embase]/lim OR

[medline]/lim)) OR ('central venous

catheterization':ab,ti AND ([embase]/lim OR

[medline]/lim)) OR ('catheterizations, central

venous':ab,ti AND ([embase]/lim OR

[medline]/lim)) OR ('central venous

catheterizations':ab,ti AND ([embase]/lim OR

[medline]/lim)) OR ('venous catheterizations,

central':ab,ti AND ([embase]/lim OR

[medline]/lim)) OR ('catheterization':ab,ti AND

([embase]/lim OR [medline]/lim)) OR

('catheterizations':ab,ti AND ([embase]/lim OR

[medline]/lim)) OR ('cannulation':ab,ti AND

([embase]/lim OR [medline]/lim)) OR

('cannulations':ab,ti AND ([embase]/lim OR

[medline]/lim)) OR ('catheters, indwelling':ab,ti

AND ([embase]/lim OR [medline]/lim)) OR

('catheter, indwelling':ab,ti AND ([embase]/lim

OR [medline]/lim)) OR ('indwelling

catheter':ab,ti AND ([embase]/lim OR

[medline]/lim)) OR ('indwelling catheters':ab,ti

AND ([embase]/lim OR [medline]/lim)) OR

('in-dwelling catheters':ab,ti AND ([embase]/lim

OR [medline]/lim)) OR ('catheter,

in-dwelling':ab,ti AND ([embase]/lim OR

[medline]/lim)) OR ('catheters,

in-dwelling':ab,ti AND ([embase]/lim OR

[medline]/lim)) OR ('in dwelling catheters':ab,ti

AND ([embase]/lim OR [medline]/lim)) OR

('in-dwelling catheter':ab,ti AND ([embase]/lim

OR [medline]/lim)) OR ('implantable

catheters':ab,ti AND ([embase]/lim OR

[medline]/lim)) OR ('catheter*':ab,ti AND

([embase]/lim OR [medline]/lim)) OR

('cannula*':ab,ti AND ([embase]/lim OR

[medline]/lim)) OR ('cvc*':ab,ti AND

([embase]/lim OR [medline]/lim)) OR ('totally

implanted venous access devices':ab,ti AND

([embase]/lim OR [medline]/lim)) OR ('totally

implanted venous access device':ab,ti AND

([embase]/lim OR [medline]/lim)) OR

('tivad*':ab,ti AND ([embase]/lim OR

[medline]/lim)) OR ('venous access device':ab,ti

AND ([embase]/lim OR [medline]/lim)) OR

('peripherally inserted central catheter line

insertion':ab,ti AND ([embase]/lim OR

[medline]/lim)) OR ('picc placement':ab,ti AND

([embase]/lim OR [medline]/lim)) OR ('picc

placements':ab,ti AND ([embase]/lim OR

[medline]/lim)) OR ('placement, picc':ab,ti AND

([embase]/lim OR [medline]/lim)) OR ('placements,

picc':ab,ti AND ([embase]/lim OR [medline]/lim))

OR ('picc line placement':ab,ti AND ([embase]/lim

OR [medline]/lim)) OR ('picc line

placements':ab,ti AND ([embase]/lim OR

[medline]/lim)) OR ('placement, picc line':ab,ti

AND ([embase]/lim OR [medline]/lim)) OR

('placements, picc line':ab,ti AND ([embase]/lim

OR [medline]/lim)) OR ('picc line

catheterization':ab,ti AND ([embase]/lim OR

[medline]/lim)) OR ('catheterization, picc

line':ab,ti AND ([embase]/lim OR [medline]/lim))

OR ('catheterizations, picc line':ab,ti AND

([embase]/lim OR [medline]/lim)) OR ('picc line

catheterizations':ab,ti AND ([embase]/lim OR

[medline]/lim)) OR ('peripherally inserted

central catheters':ab,ti AND ([embase]/lim OR

[medline]/lim)) OR ('peripherally inserted

central catheter':ab,ti AND ([embase]/lim OR

[medline]/lim)) OR ('picc*':ab,ti AND

([embase]/lim OR [medline]/lim)) OR 'central

venous catheter'/exp OR 'peripherally inserted

central venous catheter'/exp OR 'central venous

catheterization'/exp) AND ('randomized controlled

trial'/exp OR 'randomized controlled trial

(topic)'/exp OR ('randomized controlled

trial':ab,ti AND ([embase]/lim OR [medline]/lim))

OR ('random*':ab,ti AND ([embase]/lim OR

[medline]/lim)))

#97. 'randomized controlled trial'/exp OR 'randomized **1,254,638** 28 Sep 2016

controlled trial (topic)'/exp OR ('randomized

controlled trial':ab,ti AND ([embase]/lim OR

[medline]/lim)) OR ('random*':ab,ti AND

([embase]/lim OR [medline]/lim))

#96. 'random*':ab,ti AND ([embase]/lim OR 1,109,366 28 Sep 2016

[medline]/lim)

#95. 'randomized controlled trial':ab,ti AND 59,285 28 Sep 2016

([embase]/lim OR [medline]/lim)

#94. 'randomized controlled trial (topic)'/exp 104,287 28 Sep 2016

#93. 'randomized controlled trial'/exp 414,081 28 Sep 2016

#92. 'catheterization, central venous':ab,ti AND **298,212** 28 Sep 2016

([embase]/lim OR [medline]/lim) OR ('venous

catheterization, central':ab,ti AND ([embase]/lim

OR [medline]/lim)) OR ('central

catheterization':ab,ti AND ([embase]/lim OR

[medline]/lim)) OR ('catheterization,

central':ab,ti AND ([embase]/lim OR

[medline]/lim)) OR ('catheterizations,

central':ab,ti AND ([embase]/lim OR

[medline]/lim)) OR ('central

catheterizations':ab,ti AND ([embase]/lim OR

[medline]/lim)) OR ('central venous

catheterization':ab,ti AND ([embase]/lim OR

[medline]/lim)) OR ('catheterizations, central

venous':ab,ti AND ([embase]/lim OR

[medline]/lim)) OR ('central venous

catheterizations':ab,ti AND ([embase]/lim OR

[medline]/lim)) OR ('venous catheterizations,

central':ab,ti AND ([embase]/lim OR

[medline]/lim)) OR ('catheterization':ab,ti AND

([embase]/lim OR [medline]/lim)) OR

('catheterizations':ab,ti AND ([embase]/lim OR

[medline]/lim)) OR ('cannulation':ab,ti AND

([embase]/lim OR [medline]/lim)) OR

('cannulations':ab,ti AND ([embase]/lim OR

[medline]/lim)) OR ('catheters, indwelling':ab,ti

AND ([embase]/lim OR [medline]/lim)) OR

('catheter, indwelling':ab,ti AND ([embase]/lim

OR [medline]/lim)) OR ('indwelling

catheter':ab,ti AND ([embase]/lim OR

[medline]/lim)) OR ('indwelling catheters':ab,ti

AND ([embase]/lim OR [medline]/lim)) OR

('in-dwelling catheters':ab,ti AND ([embase]/lim

OR [medline]/lim)) OR ('catheter,

in-dwelling':ab,ti AND ([embase]/lim OR

[medline]/lim)) OR ('catheters,

in-dwelling':ab,ti AND ([embase]/lim OR

[medline]/lim)) OR ('in dwelling catheters':ab,ti

AND ([embase]/lim OR [medline]/lim)) OR

('in-dwelling catheter':ab,ti AND ([embase]/lim

OR [medline]/lim)) OR ('implantable

catheters':ab,ti AND ([embase]/lim OR

[medline]/lim)) OR ('catheter*':ab,ti AND

([embase]/lim OR [medline]/lim)) OR

('cannula*':ab,ti AND ([embase]/lim OR

[medline]/lim)) OR ('cvc*':ab,ti AND

([embase]/lim OR [medline]/lim)) OR ('totally

implanted venous access devices':ab,ti AND

([embase]/lim OR [medline]/lim)) OR ('totally

implanted venous access device':ab,ti AND

([embase]/lim OR [medline]/lim)) OR

('tivad*':ab,ti AND ([embase]/lim OR

[medline]/lim)) OR ('venous access device':ab,ti

AND ([embase]/lim OR [medline]/lim)) OR

('peripherally inserted central catheter line

insertion':ab,ti AND ([embase]/lim OR

[medline]/lim)) OR ('picc placement':ab,ti AND

([embase]/lim OR [medline]/lim)) OR ('picc

placements':ab,ti AND ([embase]/lim OR

[medline]/lim)) OR ('placement, picc':ab,ti AND

([embase]/lim OR [medline]/lim)) OR ('placements,

picc':ab,ti AND ([embase]/lim OR [medline]/lim))

OR ('picc line placement':ab,ti AND ([embase]/lim

OR [medline]/lim)) OR ('picc line

placements':ab,ti AND ([embase]/lim OR

[medline]/lim)) OR ('placement, picc line':ab,ti

AND ([embase]/lim OR [medline]/lim)) OR

('placements, picc line':ab,ti AND ([embase]/lim

OR [medline]/lim)) OR ('picc line

catheterization':ab,ti AND ([embase]/lim OR

[medline]/lim)) OR ('catheterization, picc

line':ab,ti AND ([embase]/lim OR [medline]/lim))

OR ('catheterizations, picc line':ab,ti AND

([embase]/lim OR [medline]/lim)) OR ('picc line

catheterizations':ab,ti AND ([embase]/lim OR

[medline]/lim)) OR ('peripherally inserted

central catheters':ab,ti AND ([embase]/lim OR

[medline]/lim)) OR ('peripherally inserted

central catheter':ab,ti AND ([embase]/lim OR

[medline]/lim)) OR ('picc*':ab,ti AND

([embase]/lim OR [medline]/lim)) OR 'central

venous catheter'/exp OR 'peripherally inserted

central venous catheter'/exp OR 'central venous

catheterization'/exp

#91. 'central venous catheterization'/exp 7,748 28 Sep 2016

#90. 'peripherally inserted central venous 1,762 28 Sep 2016

catheter'/exp

#89. 'central venous catheter'/exp 17,415 28 Sep 2016

#88. 'picc*':ab,ti AND ([embase]/lim OR [medline]/lim) 4,575 28 Sep 2016

#87. 'peripherally inserted central catheter':ab,ti 741 28 Sep 2016

AND ([embase]/lim OR [medline]/lim)

#86. 'peripherally inserted central catheters':ab,ti 812 28 Sep 2016

AND ([embase]/lim OR [medline]/lim)

#85. 'picc line catheterizations':ab,ti AND 28 Sep 2016

([embase]/lim OR [medline]/lim)

#84. 'catheterizations, picc line':ab,ti AND 28 Sep 2016

([embase]/lim OR [medline]/lim)

#83. 'catheterization, picc line':ab,ti AND 28 Sep 2016

([embase]/lim OR [medline]/lim)

#82. 'picc line catheterization':ab,ti AND 28 Sep 2016

([embase]/lim OR [medline]/lim)

#81. 'placements, picc line':ab,ti AND ([embase]/lim 28 Sep 2016

OR [medline]/lim)

#80. 'placement, picc line':ab,ti AND ([embase]/lim OR 28 Sep 2016

[medline]/lim)

#79. 'picc line placements':ab,ti AND ([embase]/lim OR 7 28 Sep 2016

[medline]/lim)

#78. 'picc line placement':ab,ti AND ([embase]/lim OR 37 28 Sep 2016

[medline]/lim)

#77. 'placements, picc':ab,ti AND ([embase]/lim OR 28 Sep 2016

[medline]/lim)

#76. 'placement, picc':ab,ti AND ([embase]/lim OR 28 Sep 2016

[medline]/lim)

#75. 'picc placements':ab,ti AND ([embase]/lim OR 38 28 Sep 2016

[medline]/lim)

#74. 'picc placement':ab,ti AND ([embase]/lim OR 199 28 Sep 2016

[medline]/lim)

#73. 'peripherally inserted central catheter line 1 28 Sep 2016

insertion':ab,ti AND ([embase]/lim OR

[medline]/lim)

#72. 'venous access device':ab,ti AND ([embase]/lim OR 471 28 Sep 2016

[medline]/lim)

#71. 'tivad*':ab,ti AND ([embase]/lim OR 147 28 Sep 2016

[medline]/lim)

#70. 'totally implanted venous access device':ab,ti 6 28 Sep 2016

AND ([embase]/lim OR [medline]/lim)

#69. 'totally implanted venous access devices':ab,ti 17 28 Sep 2016

AND ([embase]/lim OR [medline]/lim)

#68. 'cvc*':ab,ti AND ([embase]/lim OR [medline]/lim) 5,744 28 Sep 2016

#67. 'cannula*':ab,ti AND ([embase]/lim OR 48,739 28 Sep 2016

[medline]/lim)

#66. 'catheter*':ab,ti AND ([embase]/lim OR 244,240 28 Sep 2016

[medline]/lim)

#65. 'implantable catheters':ab,ti AND ([embase]/lim 50 28 Sep 2016

OR [medline]/lim)

#64. 'in-dwelling catheter':ab,ti AND ([embase]/lim OR 49 28 Sep 2016

[medline]/lim)

#63. 'in dwelling catheters':ab,ti AND ([embase]/lim 39 28 Sep 2016

OR [medline]/lim)

#62. 'catheters, in-dwelling':ab,ti AND ([embase]/lim 2 28 Sep 2016

OR [medline]/lim)

#61. 'catheter, in-dwelling':ab,ti AND ([embase]/lim 28 Sep 2016

OR [medline]/lim)

#60. 'in-dwelling catheters':ab,ti AND ([embase]/lim 39 28 Sep 2016

OR [medline]/lim)

#59. 'indwelling catheters':ab,ti AND ([embase]/lim OR 1,894 28 Sep 2016

[medline]/lim)

#58. 'indwelling catheter':ab,ti AND ([embase]/lim OR 2,737 28 Sep 2016

[medline]/lim)

#57. 'catheter, indwelling':ab,ti AND ([embase]/lim OR 213 28 Sep 2016

[medline]/lim)

#56. 'catheters, indwelling':ab,ti AND ([embase]/lim 21 28 Sep 2016

OR [medline]/lim)

#55. 'cannulations':ab,ti AND ([embase]/lim OR 656 28 Sep 2016

[medline]/lim)

#54. 'cannulation':ab,ti AND ([embase]/lim OR 15,377 28 Sep 2016

[medline]/lim)

#53. 'catheterizations':ab,ti AND ([embase]/lim OR 2,432 28 Sep 2016

[medline]/lim)

#52. 'catheterization':ab,ti AND ([embase]/lim OR 60,910 28 Sep 2016

[medline]/lim)

#51. 'venous catheterizations, central':ab,ti AND 28 Sep 2016

([embase]/lim OR [medline]/lim)

#50. 'central venous catheterizations':ab,ti AND 52 28 Sep 2016

([embase]/lim OR [medline]/lim)

#49. 'catheterizations, central venous':ab,ti AND 28 Sep 2016

([embase]/lim OR [medline]/lim)

#48. 'central venous catheterization':ab,ti AND 1,429 28 Sep 2016

([embase]/lim OR [medline]/lim)

#47. 'central catheterizations':ab,ti AND 7 28 Sep 2016

([embase]/lim OR [medline]/lim)

#46. 'catheterizations, central':ab,ti AND 1 28 Sep 2016

([embase]/lim OR [medline]/lim)

#45. 'catheterization, central':ab,ti AND 28 28 Sep 2016

([embase]/lim OR [medline]/lim)

#44. 'central catheterization':ab,ti AND ([embase]/lim 74 28 Sep 2016

OR [medline]/lim)

#43. 'venous catheterization, central':ab,ti AND 6 28 Sep 2016

([embase]/lim OR [medline]/lim)

#42. 'catheterization, central venous':ab,ti AND 18 28 Sep 2016

([embase]/lim OR [medline]/lim)

#41. 'heparin':ab,ti AND ([embase]/lim OR **214,599** 28 Sep 2016

[medline]/lim) OR ('unfractionated heparin':ab,ti

AND ([embase]/lim OR [medline]/lim)) OR

('heparin, unfractionated':ab,ti AND

([embase]/lim OR [medline]/lim)) OR ('heparinic

acid':ab,ti AND ([embase]/lim OR [medline]/lim))

OR ('liquaemin':ab,ti AND ([embase]/lim OR

[medline]/lim)) OR ('sodium heparin':ab,ti AND

([embase]/lim OR [medline]/lim)) OR ('heparin,

sodium':ab,ti AND ([embase]/lim OR

[medline]/lim)) OR ('heparin sodium':ab,ti AND

([embase]/lim OR [medline]/lim)) OR

('alpha-heparin':ab,ti AND ([embase]/lim OR

[medline]/lim)) OR ('alpha heparin':ab,ti AND

([embase]/lim OR [medline]/lim)) OR

('hepar*':ab,ti AND ([embase]/lim OR

[medline]/lim)) OR ('uh':ab,ti AND ([embase]/lim

OR [medline]/lim)) OR ('ufh':ab,ti AND

([embase]/lim OR [medline]/lim)) OR ('lmwh':ab,ti

AND ([embase]/lim OR [medline]/lim)) OR

('hs':ab,ti AND ([embase]/lim OR [medline]/lim))

OR 'heparin'/exp

#40. 'heparin'/exp 131,682 28 Sep 2016

#39. 'hs':ab,ti AND ([embase]/lim OR [medline]/lim) 35,840 28 Sep 2016

#38. 'lmwh':ab,ti AND ([embase]/lim OR [medline]/lim) 6,936 28 Sep 2016

#37. 'ufh':ab,ti AND ([embase]/lim OR [medline]/lim) 2,973 28 Sep 2016

#36. 'uh':ab,ti AND ([embase]/lim OR [medline]/lim) 1,318 28 Sep 2016

#35. 'hepar*':ab,ti AND ([embase]/lim OR 111,435 28 Sep 2016

[medline]/lim)

#34. 'alpha heparin':ab,ti AND ([embase]/lim OR 10 28 Sep 2016

[medline]/lim)

#33. 'alpha-heparin':ab,ti AND ([embase]/lim OR 10 28 Sep 2016

[medline]/lim)

#32. 'heparin sodium':ab,ti AND ([embase]/lim OR 578 28 Sep 2016

[medline]/lim)

#31. 'heparin, sodium':ab,ti AND ([embase]/lim OR 578 28 Sep 2016

[medline]/lim)

#30. 'sodium heparin':ab,ti AND ([embase]/lim OR 455 28 Sep 2016

[medline]/lim)

#29. 'liquaemin':ab,ti AND ([embase]/lim OR 7 28 Sep 2016

[medline]/lim)

#28. 'heparinic acid':ab,ti AND ([embase]/lim OR 4 28 Sep 2016

[medline]/lim)

#27. 'heparin, unfractionated':ab,ti AND ([embase]/lim 86 28 Sep 2016

OR [medline]/lim)

#26. 'unfractionated heparin':ab,ti AND ([embase]/lim 7,083 28 Sep 2016

OR [medline]/lim)

#25. 'heparin':ab,ti AND ([embase]/lim OR 89,316 28 Sep 2016

[medline]/lim)

#24. 'saline solution, hypertonic':ab,ti AND **709,528** 28 Sep 2016

([embase]/lim OR [medline]/lim) OR ('hypertonic

solutions, saline':ab,ti AND ([embase]/lim OR

[medline]/lim)) OR ('saline hypertonic

solutions':ab,ti AND ([embase]/lim OR

[medline]/lim)) OR ('solutions, saline

hypertonic':ab,ti AND ([embase]/lim OR

[medline]/lim)) OR ('saline solutions,

hypertonic':ab,ti AND ([embase]/lim OR

[medline]/lim)) OR ('sodium chloride solution,

hypertonic':ab,ti AND ([embase]/lim OR

[medline]/lim)) OR ('hypertonic solution,

saline':ab,ti AND ([embase]/lim OR

[medline]/lim)) OR ('saline hypertonic

solution':ab,ti AND ([embase]/lim OR

[medline]/lim)) OR ('solution, saline

hypertonic':ab,ti AND ([embase]/lim OR

[medline]/lim)) OR ('sodium chloride solutions,

hypertonic':ab,ti AND ([embase]/lim OR

[medline]/lim)) OR ('hypertonic saline

solutions':ab,ti AND ([embase]/lim OR

[medline]/lim)) OR ('solutions, hypertonic

saline':ab,ti AND ([embase]/lim OR

[medline]/lim)) OR ('hypertonic saline

solution':ab,ti AND ([embase]/lim OR

[medline]/lim)) OR ('solution, hypertonic

saline':ab,ti AND ([embase]/lim OR

[medline]/lim)) OR ('sodium chloride':ab,ti AND

([embase]/lim OR [medline]/lim)) OR ('saline

solution':ab,ti AND ([embase]/lim OR

[medline]/lim)) OR ('sodium chloride,

(22)na':ab,ti AND ([embase]/lim OR

[medline]/lim)) OR ('sodium chloride,

(24)nacl':ab,ti AND ([embase]/lim OR

[medline]/lim)) OR ('saline*':ab,ti AND

([embase]/lim OR [medline]/lim)) OR

('sodium':ab,ti AND ([embase]/lim OR

[medline]/lim)) OR ('nacl':ab,ti AND

([embase]/lim OR [medline]/lim)) OR ('ns':ab,ti

AND ([embase]/lim OR [medline]/lim)) OR 'sodium

chloride'/exp

#23. 'sodium chloride'/exp 154,434 28 Sep 2016

#22. 'ns':ab,ti AND ([embase]/lim OR [medline]/lim) 81,093 28 Sep 2016

#21. 'nacl':ab,ti AND ([embase]/lim OR [medline]/lim) 60,533 28 Sep 2016

#20. 'sodium':ab,ti AND ([embase]/lim OR 360,991 28 Sep 2016

[medline]/lim)

#19. 'saline*':ab,ti AND ([embase]/lim OR 193,743 28 Sep 2016

[medline]/lim)

#18. 'sodium chloride, (24)nacl':ab,ti AND 28 Sep 2016

([embase]/lim OR [medline]/lim)

#17. 'sodium chloride, (22)na':ab,ti AND ([embase]/lim 28 Sep 2016

OR [medline]/lim)

#16. 'saline solution':ab,ti AND ([embase]/lim OR 15,883 28 Sep 2016

[medline]/lim)

#15. 'sodium chloride':ab,ti AND ([embase]/lim OR 17,304 28 Sep 2016

[medline]/lim)

#14. 'solution, hypertonic saline':ab,ti AND 11 28 Sep 2016

([embase]/lim OR [medline]/lim)

#13. 'hypertonic saline solution':ab,ti AND 589 28 Sep 2016

([embase]/lim OR [medline]/lim)

#12. 'solutions, hypertonic saline':ab,ti AND 3 28 Sep 2016

([embase]/lim OR [medline]/lim)

#11. 'hypertonic saline solutions':ab,ti AND 198 28 Sep 2016

([embase]/lim OR [medline]/lim)

#10. 'sodium chloride solutions, hypertonic':ab,ti AND 28 Sep 2016

([embase]/lim OR [medline]/lim)

#9. 'solution, saline hypertonic':ab,ti AND 28 Sep 2016

([embase]/lim OR [medline]/lim)

#8. 'saline hypertonic solution':ab,ti AND 5 28 Sep 2016

([embase]/lim OR [medline]/lim)

#7. 'hypertonic solution, saline':ab,ti AND 28 Sep 2016

([embase]/lim OR [medline]/lim)

#6. 'sodium chloride solution, hypertonic':ab,ti AND 2 28 Sep 2016

([embase]/lim OR [medline]/lim)

#5. 'saline solutions, hypertonic':ab,ti AND 28 Sep 2016

([embase]/lim OR [medline]/lim)

#4. 'solutions, saline hypertonic':ab,ti AND 28 Sep 2016

([embase]/lim OR [medline]/lim)

#3. 'saline hypertonic solutions':ab,ti AND 1 28 Sep 2016

([embase]/lim OR [medline]/lim)

#2. 'hypertonic solutions, saline':ab,ti AND 28 Sep 2016

([embase]/lim OR [medline]/lim)

#1. 'saline solution, hypertonic':ab,ti AND 3 28 Sep 2016

([embase]/lim OR [medline]/lim)

.......................................................
