# Supplementary material for: Normal saline versus heparin for patency of central venous catheters in adult patients - a systematic review and meta-analysis
Source: Crit Care. 2017 Jan 8;21:5. doi: 10.1186/s13054-016-1585-x (PMC5219914; doi:10.1186/s13054-016-1585-x)
Supplement: Additional file 4: — Characteristics of the three relevant meta-analyses. (DOCX 37 kb) [file 13054_2016_1585_MOESM4_ESM.docx]

**Additional file 4:** **is a table presenting characteristics of the three relevant meta-analyses**

| **Study Patients** **Intervention Comparison Outcomes** |
| --- |
| Molin 2014 [[1](#_ENREF_1)] Adult patients HS HS or Obstructions, infections, venous thrombosis, heparin induced  other solution thrombocytopenia, other complications related to the management  of central venous catheter.  López-Briz 2014 [[2](#_ENREF_2)] Adult patients HS NS Occlusion, duration of catheter patency, CVC-related sepsis,  mortality, Haemorrhage at any site.  Santos 2015 [[3](#_ENREF_3)] Adult patients HS NS Occlusion  Our study Adult patients NS HS Catheter occlusion, maneuver needed, heparin-induced  thrombocytopenia, haemorrhage, central venous thrombosis,  catheter-related bloodstream infection |

| **Study Study design Results(Occlusions) Conclusion** |
| --- |
| Molin 2014 [[1](#_ENREF_1)] HS vs. NS: Five RCTs studies Pooled RR: 0.55 [0.12, 1.37] There is no evidence of a different effectiveness  HS vs. other solutions: Four RCTs studies between heparin flushing and normal saline or  other solutions in reducing catheter occlusions.  López-Briz 2014 [[2](#_ENREF_2)] Six RCTs studies For participant: RR 0.21 [0.03, 1.70]; HS vs. NS: no statistically significant;  For catheter: RR 0.53 [0.29, 0.94]; As heparin is more expensive than normal saline,  For line access: RR 1.08 [0.8, 1.40]. our findings challenge its continued use in CVC  flushing outside the context of clinical trials .  Santos 2015 [[3](#_ENREF_3)] Seven RCTs studies For fully deployed CVC: RR 1.09 [0.53, 2.22];  HS vs. NS: no statistically significant;  For multiple-Lumen CVC: RR 0.53 [0.29, 0.95]; Saline solution is sufficient for maintaining  For double-Lumen CVC: RR 1.18 [0.08, 17.82]; patency of the central venous catheter,  For Peripherally inserted CVC: RR 0.14 [0.01, 2.60]. preventing the risks associated with heparin  Pooled RR: 0.68 [0.41, 1.10] administration.  Our study Ten RCTs studies See Figure 4, 5 and 6 in our manuscript HS vs. NS: no statistically significant;  Based on the results of this meta-analysis, HS is not  superior to NS in reducing CVCs occlusion. But in  the short term, the use of HS is slightly superior  to NS for flushing catheters from a statistical point  of view. |

Compared with previous meta-analyses, our study provides some additional information: the addition of three studies, an increase in the number of patients examined, Table 2 in our manuscript contains more detailed reading, etc.

Simultaneously, several specific statistical methods (funnel plot and sensitivity analysis) were first used to evaluate publication bias and reporting biases of included studies.

To date, there have only been three relevant meta-analyses in this area. The result of the first study (network meta-analysis) was no marked difference, comparing adult patients with NS versus HS or other solutions in the flushing of CVCs [[1](#_ENREF_1)]. The second study, consistent with result of previous research, found that HS was not more effective than NS in reducing catheter occlusion in three different areas (participant, catheter and line access). Their findings challenged the continued use of HS in CVCs flushing as it is more expensive than saline solution [[2](#_ENREF_2)]. However, a recent study supported that NS is regarded as a substitution for HS as a locking solution in CVCs in adult patients from the point of view of four different CVCs types [[3](#_ENREF_3)].

It is noteworthy that this might be the first meta-analysis which shows that flushing CVCs with NS is as effective as HS in adult patients from the viewpoint of four different CVCs related areas (patient, catheter, lumen and line access) as well as indwelling time (i.e. <30 and >30 days).

From the point of four different units, we discover that they impart us more revelation and make us view many issues of CVCs much more.

**References**

1. Dal Molin A, Allara E, Montani D, Milani S, Frassati C, Cossu S, Tonella S, Brioschi D, Rasero L: **Flushing the central venous catheter: Is heparin necessary?** *Journal of Vascular Access* 2014, **15**(4):241-248.

2. López-Briz E, Ruiz Garcia V, Cabello JB, Bort-Marti S, Carbonell Sanchis R, Burls A: **Heparin versus 0.9% sodium chloride intermittent flushing for prevention of occlusion in central venous catheters in adults**. *The Cochrane database of systematic reviews* 2014, **10**:CD008462.

3. Santos EJ, Nunes MM, Cardoso DF, Apostolo JL, Queiros PJ, Rodrigues MA: **[Effectiveness of heparin versus 0.9% saline solution in maintaining the permeability of central venous catheters: a systematic review]**. *Revista da Escola de Enfermagem da U S P* 2015, **49**(6):999-1007.
